# Technology for Repairing Osteomyelitic Bone Defects Using Autologous Mesenchymal Stromal Cells on a Collagen Matrix in Experiment

**DOI:** 10.17691/stm2021.13.1.05

**Published:** 2021-02-28

**Authors:** V.N. Mitrofanov, O.P. Zhivtsov, N.Yu. Orlinskaya, D.V. Davydenko, I.N. Charykova, D.Ya. Aleinik

**Affiliations:** Head of the Department of Septic Surgery, University Clinic, Privolzhsky Research Medical University, 10/1 Minin and Pozharsky Square, Nizhny Novgorod, 603005, Russia; Researcher, Physician of Trauma Surgery, Department of Septic Surgery, University Clinic, Privolzhsky Research Medical University, 10/1 Minin and Pozharsky Square, Nizhny Novgorod, 603005, Russia; Professor, Head of the Department of Pathological Anatomy, University Clinic, Privolzhsky Research Medical University, 10/1 Minin and Pozharsky Square, Nizhny Novgorod, 603005, Russia; Researcher, Department of Pathological Anatomy, University Clinic, Privolzhsky Research Medical University, 10/1 Minin and Pozharsky Square, Nizhny Novgorod, 603005, Russia; Physician of Clinical Laboratory Diagnostics, Laboratory of Biotechnology, University Clinic, Privolzhsky Research Medical University, 10/1 Minin and Pozharsky Square, Nizhny Novgorod, 603005, Russia; Senior Researcher, Research Institute of Experimental Oncology and Biomedical Technologies, Privolzhsky Research Medical University, 10/1 Minin and Pozharsky Square, Nizhny Novgorod, 603005, Russia

**Keywords:** chronic osteomyelitis, mesenchymal stem cells, repair of bone defects, regenerative technologies

## Abstract

**The aim of the study** was to develop a technology for repairing an osteomyelitic bone defect using autologous adipose tissue mesenchymal stromal cells (MSCs) bound to a collagen matrix and to test the efficacy of this technique.

**Materials and Methods.:**

The study was carried out with 17 rabbits. A bone defect was created using a milling cutter applied to the proximal third of the leg. The wound (8.0×4.0 mm and a depth of 4.0 mm) involved the periosteum, cortical layer, and cancellous substance. *Staphylococcus aureus* strain was used as an infectious agent.

After the development of chronic osteomyelitis, the animals underwent osteonecrectomy. In the study group, autologous MSCs in Collatamp EG collagen carrier were placed into the bone defect. MSCs were obtained from adipose tissue and cultured in the matrix for 5 days. In control, the defect was filled with the collagen matrix without cells.

**Results.:**

On day 14 upon the initiation of chronic osteomyelitis, bacteriological examination of the discharge from the fistula showed the presence of mixed bacterial flora (*Staphylococcus aureus* and *Escherichia coli*) in all operated animals. Results of X-ray, laboratory, and histological tests confirmed the formation of a focus of chronic osteomyelitis.

Two months after the treatment (collagen with or without MSCs) began, all animals of the study group showed mature bone tissue regenerated in the affected zone. In the control group, proliferation of osteoblasts on the surface of the bone trabeculae was also observed; however, mature osteoid tissue was more often detected in the study group (35.0 vs 20.0% in control). In the study group (MSCs + collagen matrix), there was a decrease in bone marrow fibrosis (50.0 vs 100.0% in control) and cartilage formation (30.0 and 66.7%, respectively). After full treatment, newly formed bone trabeculae were detected more often (100.0 vs 60.0% in control); they were more mature and filled the defect area more efficiently.

**Conclusion.:**

Our results indicate that the use of a collagen matrix with autologous MSCs is a promising plastic material for repairing osteomyelitic defects following necrectomy.

The MSCs were able to increase the density of the filling material in the bone cavity, significantly accelerate the formation of bone beams around the matrix, and increase the tissue volume around the implant. The presence of MSCs significantly decreased the interference of a connective tissue component with osteogenesis and chondrogenesis.

## Introduction

One of the current tasks in traumatology and orthopedics is the search for osteoplastic material with osteoconduction and osteoinduction effects on tissues exposed to a purulent process [1–10]. Today, cellular technologies are increasingly used in clinical situations associated with failed tissue repair. Due to their specific properties, mesenchymal stromal cells (MSCs) are considered the most promising material for regenerative medicine. Technologies for obtaining MSCs from various tissues of an adult body have been developed; currently, cells isolated from the bone marrow, periosteum, and adipose tissue are most commonly studied. It is known that adipose tissue MSCs and bone marrow MSCs are capable of osteogenic, chondrogenic, and adipogenic differentiation. De Ugarte et al. [11] found no significant osteogenic differences between cells isolated from these three tissues. Other researchers though [12, 13] demonstrated a higher osteogenic potential of bone marrow and periosteum MSCs compared to adipose tissue MSCs. However, it is only from adipose tissue that autologous cellular material can be obtained using minimally invasive procedures.

The use of autologous cells minimizes the risk of infection, simplifies the search and selection of donors, and eliminates ethical problems. The availability and relative abundance of adipose tissue MSCs, in contrast to bone marrow cells, promotes an increasing interest in their clinical application as a source of cellular material for bone regeneration [14, 15]. In animal studies, sizable restoration of bone tissue from adipose tissue MSCs has been demonstrated [16, 17].

In addition, the osteogenic potential of these cells could be improved during their cultivation *in vitro* in the presence of chemical (ascorbate and dexamethasone) or mechanical factors [18]. Thus, the availability of adipose tissue as a source of MSCs and the possibility of enhancing their osteogenic potential at the preparation stage, provide the rationale for using the adipose tissue MSCs in clinical practice.

Previously, we demonstrated the efficacy of repairing experimental bone defects in rabbits using a construct based on the Collatamp EG collagen matrix and containing allogeneic MSCs [19]. A much more difficult and long-term task is to restore the integrity of bone defects in chronic osteomyelitis with purulent-destructive foci of infection.

Bone defects in osteomyelitis are often large-sized, which increases the need for a suitable osteoplastic material [20, 21].

Implementing the methods of regenerative medicine in the treatment of chronic osteomyelitis requires a surgical technology adjusted for using MSCs in this specific disease.

**The aim of the study** was to develop a technology for repairing an osteomyelitic bone defect using autologous adipose tissue mesenchymal stromal cells bound to a collagen matrix and to test the efficacy of this technique.

## Materials and Methods

The study included 17 gray giant rabbits, 6–8 months old, weighing 1800–2100 g. The work was approved by the Ethics Committee of the Privolzhsky Research Medical University (Nizhny Novgorod, Russia). The animals were divided into 2 groups: study group (n=10) and control (n=7).

### Technique for simulating primary chronic osteomyelitis.

The model was developed in the Department of Experimental Surgery with the animal facility of the Privolzhsky Research Medical University. The study was conducted in accordance with the requirements of the European Convention for the Protection of Vertebrate Animals Used for Experimental and Other Scientific Purposes (Strasbourg, 2006). All manipulations were performed in accordance with the Order of the Ministry of Health and Social Development of the Russian Federation No.708n dated 23.08.2010 “On the approval of the rules of laboratory practice”. The animals were euthanized by air embolism under anesthesia.

*Staphylococcus aureus* strain was used as an infectious agent. All procedures and operations were performed under anesthesia (Zoletil 50 — 10 mg/kg + Xyla — 50 mg/kg).

Surgery was performed along the anterior surface of the proximal third of the leg. A milling cutter was applied to the bone to induce a defect (measuring 8×4 mm and a depth of 4 mm) in the periosteum, the cortical and cancellous layers. The wound was infected using a 24-hour culture of staphylococcus bacteria suspended in saline at a dose of 40–45 million colony-forming units (CFU) and 0.2 g of sterile quartz sand per 1 kg of animal body weight. The operation was completed by imposing a layered interrupted suture on the wound.

**Figure 1:**
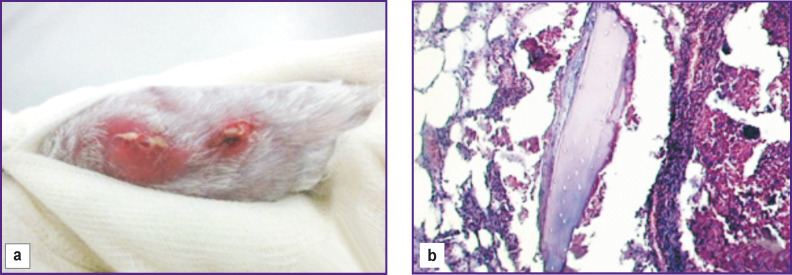
Simulated chronic osteomyelitis in a rabbit: (a) appearance; (b) histology

In order to bring the purulent inflammation into a chronic phase, the bone cavity was re-infected with a culture of *Staphylococcus aureus* at a dose of 15–20 million CFU per 1 kg of body mass; this manipulation was performed simultaneously with the fistula formation. Such “extra-inoculations” were carried out three times with intervals of 72 h. The sutures were removed from the skin 10 days after surgery (Figure 1 (a)). Bacteriological examination of the discharge from the fistula was carried out on day 14 after the operation. Mixed bacterial flora (*Staphylococcus aureus* and *Escherichia coli*) was found in all animals.

Eosinophilic plasma and lymphoid cells were found accumulating around the developing abscess. In the focus of inflammation, there were scattered bone beams containing no osteocytes (Figure 1 (b)).

The results of X-ray scan, laboratory and histological tests confirmed the formation of a focus of chronic osteomyelitis.

### Surgical debridement and implantation of the replacement material.

**Figure 2:**
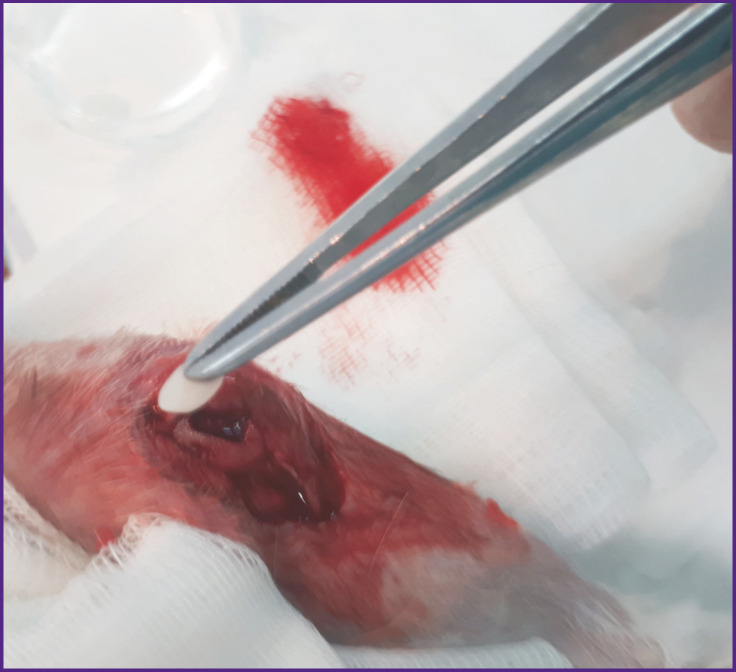
Implantation of autologous MSCs in a collagen matrix: the intra-operative stage

The animals of both groups underwent osteonecrectomy. In the study group, autologous MSCs in the Collatamp EG collagen carrier (Collatamp EG; Suntacoll GmbH, Germany) were placed in the bone defect (Figure 2); this material was found the least cytotoxic compared to other collagen carriers [22]. In animals of the control group, the defect was replaced with the collagen matrix without cells.

### X-ray examination of the bone defect.

On the day of euthanasia, the animals were examined for the size, location, and configuration of the bone defect, as well as the structure of the bone tissue. We used a DIRA-RC instrument (Roesys GmbH, Germany) for X-ray scanning in two projections and a Toshiba Aquilion 32 (Toshiba, Germany) for computed tomography. The bone density was calculated from the X-ray scans and expressed in Hounsfield units (HU).

### Preparation and cultivation of autologous MSCs.

Under sterile conditions of the animal operating room, simultaneously with the induction of a bone defect, samples of adipose tissue (0.8–1.0 cm^3^) were taken from the lumbar area of the operated rabbits. The samples were placed in test tubes filled with a transportation medium (medium 199 or Hanks’ solution added with penicillin/streptomycin) and transferred to the laboratory. Cells were isolated from adipose tissue using thermal enzymatic treatment with type 1 collagenase (PanEko, Russia) for an hour at 37°C and cultured in αMEM supplemented with 20% fetal calf serum, glutamine, and antibiotics (penicillin/streptomycin) at absolute humidity, 37°C, and 5% CO2. In this procedure, we used reagents and media from PanEko and plasticware from Costar (USA). Upon receipt, each culture was labeled according to the number of the animal. After reaching 60% confluency in a monolayer, the cells were subcultured; then, the medium was changed every 2–3 days. After 3 or 4 passages, the culture was taken for experimentation; the initial cell density was 5000/cm^2^.

The state and changes of the cultured cells were monitored using an inverted microscope Leica DMI3000 B (Leica Microsystems, Germany) combined with the LAS v. 4.3 software (Leica Microsystems). The cell phenotype was characterized using monoclonal antibodies CD44 FITC, CD105 PE, CD45 PE with the appropriate isotypic control with the help of a FACSCanto II cytofluorimeter (Becton Dickinson, USA). The results were expressed as the proportion of cells carrying the respective marker (in percent). The differentiation potential of the cells was assessed in cultures of the 3^rd^ passage. Osteogenic differentiation was induced using a mixture of differentiation factors: dexamethasone at 1 mmol/L, ascorbate diphosphate at 5 mg/ml, and glycerophosphate at 10 mM. To induce adipogenic differentiation, cells were cultured in a medium containing dexamethasone at 1 mmol/L, insulin at 2.5 mg/ml, indomethacin at 100 mmol/L, and rosiglitazone at 3.5 mmol/L. OilRed (Sigma-Aldrich, USA) was used for specific staining of lipid vacuoles; alizarin red (Sigma-Aldrich) was used to detect calcium salts during differentiation into osteoblasts and osteocalcin.

### Preparation of MSC and the matrix for administration to animals.

In our experiments both *in vitro* and *in vivo*, cells of the 3^rd^ passage were used. The cells were removed from the plastic surface using a mixture of trypsin and versene, re-suspended, collected in 1 ml syringes, and injected directly into the bulky matrix of Collatamp EG collagen sponge. The sponge samples were prepared so to adjust to the size of the newly-formed bone defect (8.0×4.0 mm and a depth of 4.0 mm). After the cell injection, the samples were placed in the complete growth medium and kept for 5 days (120 h). Some of the samples were used to assess the adhesion and viability of cells by fluorescence microscopy 48, 96, and 120 h after the injection into the matrix. To visualize the cells adhered to the matrix, intra-vital staining of nuclei was performed using Hoechst 3334 fluorochrome (BD Pharmingen, USA); this reagent is highly specific for the double-stranded DNA molecule (excitation at 377 nm, emission at 447 nm; imaging photometer Cytation 5 from BioTek, USA). Calcein fluorochrome (Calcein AM; BD Pharmingen), which stains the cytoplasm of viable cells only (excitation at 469 nm, emission at 525 nm; imaging photometer Cytation 5), was used to identify living cells and characterize their morphology within the tested material.

Forty-eight hours after the administration of cells, the samples intended for implantation were washed three times with sterile saline and transferred to the operating room.

### Histological examination.

Sampling for histological examination was carried out by carefully separating muscles from bones followed by isolating bone segments 1–1.5 cm long including the bone regeneration areas. This bone material was fixated in 10% buffered formalin solution for three days and then decalcified in Trilon-V solution (Khimreaktiv, Russia). The standard histological processing was performed using an Excelsior ES apparatus (Thermo Fisher Scientific, USA). After that, paraffin blocks were prepared using a HistoStar filling station (Thermo Fisher Scientific). Sections 4–6 μm thick were obtained with the help of a Microm HM 325 microtome (Thermo Fisher Scientific) and stained with hematoxylin and eosin using a Gemini AS staining station (Thermo Fisher Scientific). Microscopic examination was carried out with a Leica DM2500 light-optical microscope (Leica Microsystems) under ×100 or ×200 magnifications.

## Results

### Characterization of the cell culture.

**Figure 3:**
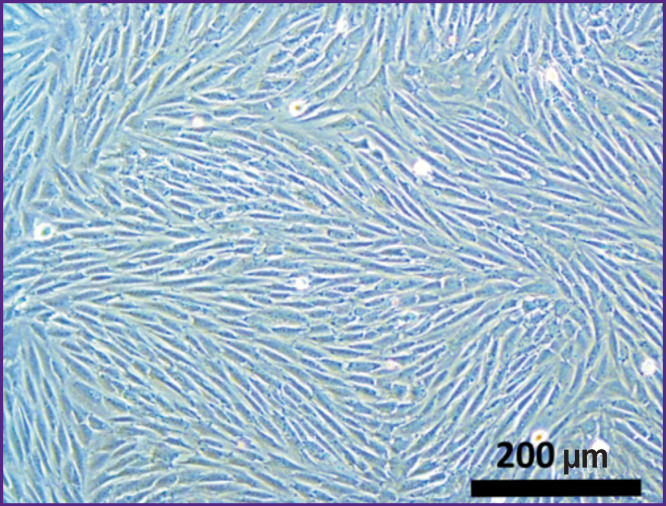
MSCs of rabbit adipose tissue before administration into the matrix; 3^rd^ passage; ×100

Cells isolated from the adipose tissue of rabbits spread well along the plastic dishes and acquired a characteristic fibroblast-like shape (Figure 3).

Throughout the entire observation period, the culture cells were morphologically homogeneous with pronounced processes and well-defined nuclei. Under the study conditions, the MSCs of the 3^rd^ passage cultures differentiated into the osteogenic and adipogenic type cells.

About 95% of these cells expressed CD44 and CD105 markers but not pan-leukocyte CD45 antigen. Therefore, the obtained cells met the criteria of human mesenchymal stem cells as defined by the International Society for Cellular Therapy [23].

### Interaction between MSCs and a xenogeneic collagen matrix.

**Figure 4:**
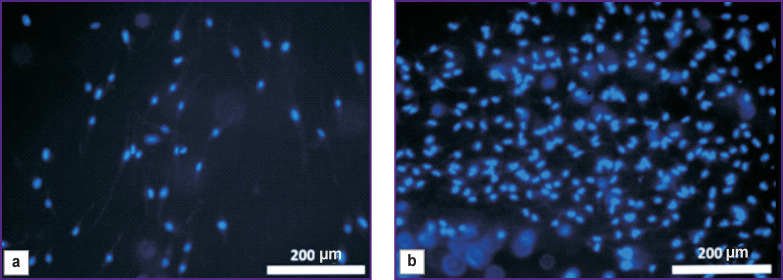
Rabbit adipose tissue MSC in the Collatamp EG matrix (staining for cell nuclei): (a) 48 h of cultivation; (b) 120 h of cultivation; fluorescence microscopy; Fluorochrome Hoechst 3334; ×100

Upon 48 h of cultivation in the Collatamp EG matrix, oval cell nuclei stained with blue were clearly visualized under fluorescence microscopy (Figure 4 (a)). Longer cultivation (96, 120 h) led to an increase in the cell density as determined by the number of stained nuclei per field of view (Figure 4 (b)).

**Figure 5:**
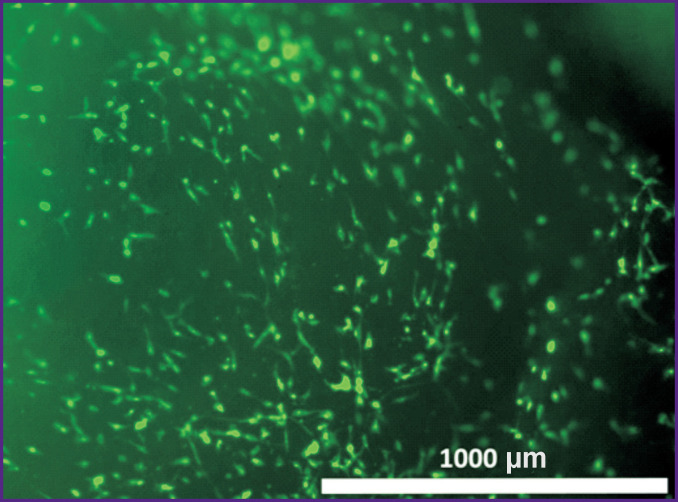
Rabbit adipose tissue MSC in the Collatamp EG matrix; fluorescence microscopy; Fluorochrome Calcein AM; 48 h of cultivation; ×40

The use of Calcein AM fluorochrome demonstrated a large number of living cells spread on the surface of the Collatamp EG matrix after 24, 48, or 120 h of cultivation (Figure 5).

From these data on cell adhesion and viability in the Collatamp EG matrix, we suggest that samples prepared the same way and used for *in vivo* experiments after 48 h, also retained viable and morphologically homogeneous MSCs.

### Clinical picture.

After surgical debridement, the process of restoration of support ability developed actively and adequately to the type of the surgery wound. After 2–3 days, the rabbits were able to use the operated limb. The wounds were healed by primary intention. The sutures were removed after 10–12 days. Neither returns of pyoinflammatory process nor development of knee contractures were observed in the animals.

### Radiographic features.

**Figure 6:**
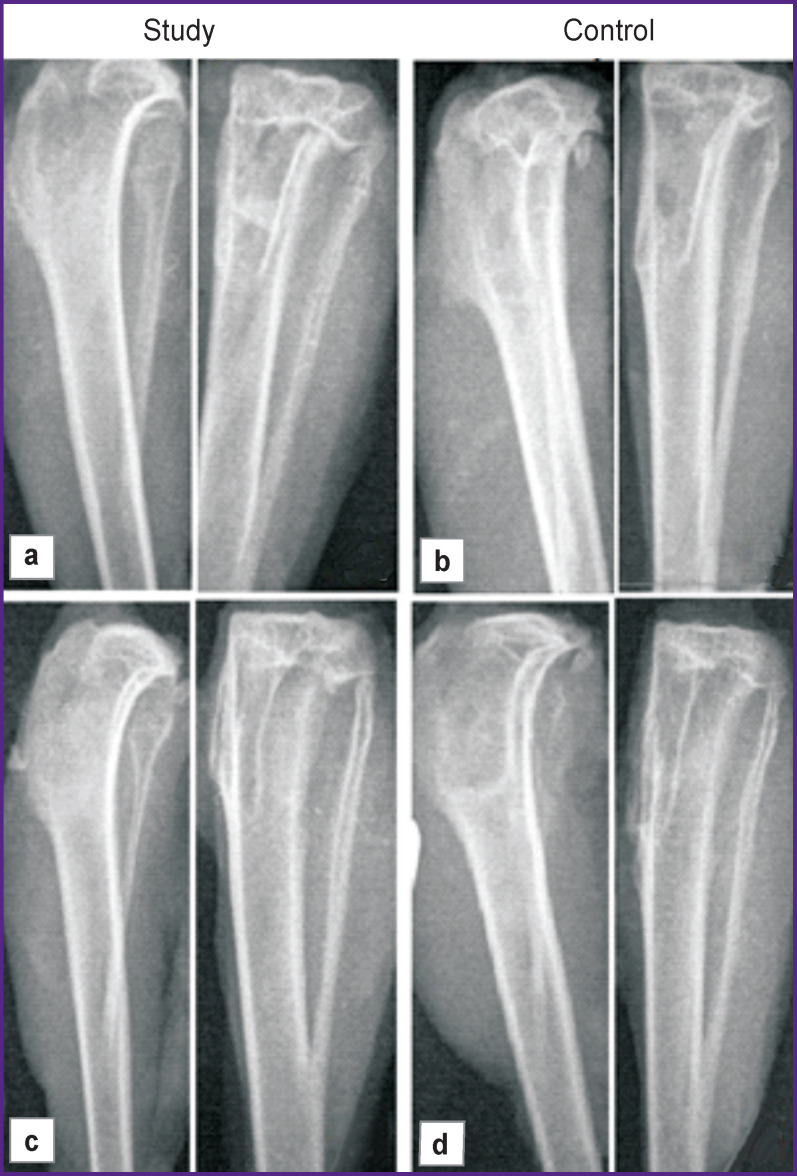
X-ray scan of the operated area: (a), (c) 30 and 60 days after implantation of autologous MSCs in the collagen matrix; (b), (d) 30 and 60 days after implantation of the collagen matrix only (control)

In rabbits of the control group, euthanized on day 30 or 60, the defect of the proximal meta-epiphysis was found to be slightly smaller than the intraoperative one. The area of lucency (the defect) was surrounded by a rim of increased radiographic density. These changes could reflect the reaction of the bone tissue to the recent osteonecrectomy and chronic inflammation. The degree of opacity of the defect zone (X-ray density) changed insignificantly, the X-ray image of the lesion was heterogeneous. There was no clear difference in the X-ray features between the 30 and 60 day subgroups (Figure 6).

In animals of the study group, after 30 days, the opacity of the lesion slightly exceeded that in the control group; the defect zone’s margins became moderately blurred (Figure 6 (a)); the opacity of the defect itself was homogenous. In animals euthanized after 60 days, the opacity of the lesion area increased significantly, its margins looked blurred even more; the size of the lesion became some smaller (Figure 6 (b)). The radiographic appearance of the lesion continued to remain homogenous and had a higher density than that in the control group. Such changes indicate the actively developing processes of bone tissue regeneration in the study group as compared with the control.

### Computed tomography (CT).

The size of the defect as per CT measurements on the day of surgery was similar to the intraoperative measurements made with a caliper. On CT densitometry, the Hounsfield index (after surgery) in the control group was 15–20 HU, which indicated the fluid nature of the cavity contents. In the cortical structures adjacent to the defect, the Hounsfield index was 680–720 HU.

In later time points, there was a decrease in the size of the defect and an increase in the average density of the replacing tissue from 184 to 224 HU.

**Figure 7:**
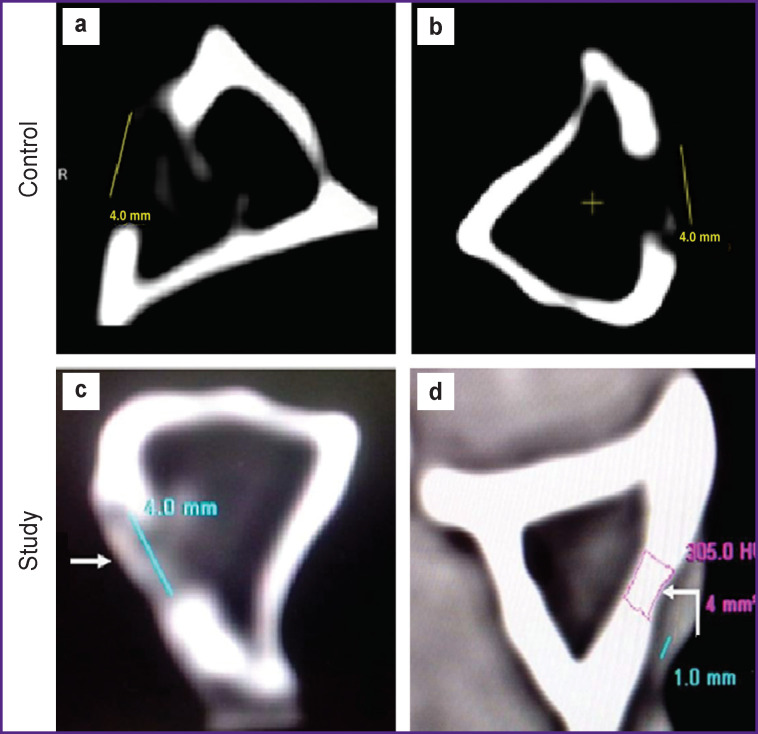
CT scan of the tibial bone defect: control group: (a) intraoperative examination, the defect is marked with the line; (b) the same area after 60 days, the line indicates the area of an incompletely restored defect; study group: (c) intraoperative examination, the defect is marked with the arrow; (d) the same area after 60 days, the arrow indicates the location of the former defect

On day 30 upon after MSC implantation, the lowest tissue density of the former defect ranged from 63 to 94 HU. When the animals were euthanized after 60 days, the lowest values of the Hounsfield index of the former defect were from 251 to 305 HU, which was close to the normal bone tissue density (Figure 7).

Thus, according to X-ray and computed tomography data, including CT densitometry, the most active processes of reparative regeneration of both the cortical defect and the bone cavity were observed when using autologous MSCs. Similar processes were described by others [24, 25], while their results may not be comparable with ours since their techniques are not clearly stated.

### Histological examination.

**Figure 8:**
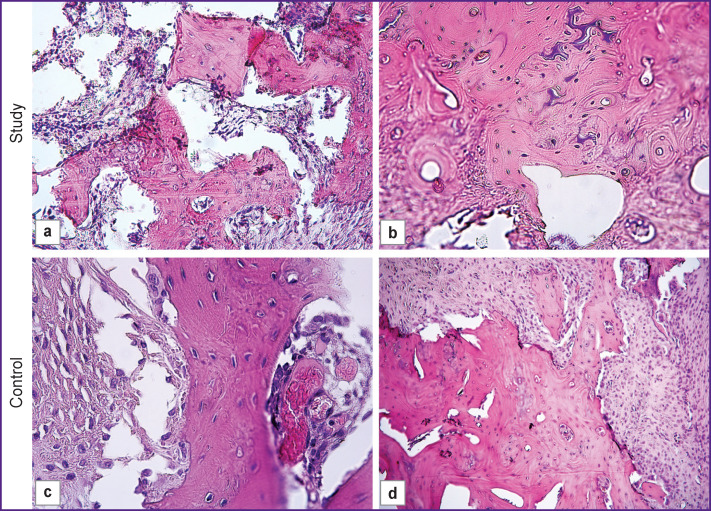
Morphology of bone tissues in the study (a), (b) and control (c), (d) groups, 30 and 60 days after the implantation surgery; staining with hematoxylin and eosin; ×200

By the end of the treatment period, the simulated bone defect was filled with osteoid tissue of varying maturity and newly formed bone trabeculae. Signs of reparative osteogenesis were found in both groups of animals. In the study group, 30 days after treatment, the defect was filled with fragments of the implant and with cells closely surrounded by newly formed bone trabeculae with proliferating osteoblasts on the surface (Figure 8 (a)). After 60 days, the formation of mature bone tissue was observed (Figure 8 (b)). The proliferation of osteoblasts on the surface of bone trabeculae was also found in the control group, however, osteoid tissues were more often detected in the study group (35.0 vs 20.0% in control). With autologous MSCs in the collagen matrix, an increased presence of newly formed bone trabeculae was noted: 100% of cases vs 70% of cases in control. In the presence of autologous MSCs, there was a decrease in bone marrow fibrosis (50.0 vs 100.0% in control) and cartilage formation (30.0 vs 66.7%) (Figure 8 (c)). Notably, osteogenesis had more beneficial features in the presence of MSCs. Thus, in animals of the study group, the newly formed bone trabeculae were more mature and more efficiently filled the defect area (100.0 vs 60% in control) (see Figure 8 (b)). The present histological results are, to some extent, comparable with reports by others, although those were mostly related to acute aseptic experiments [13, 16, 17, 24, 26].

## Conclusion

Our results indicate that using a collagen matrix with autologous mesenchymal stromal cells for filling osteomyelitic bone defects following necrectomy is promising.

The presence of mesenchymal stromal cells in a collagen matrix increases the filling density of the bone replacement material, significantly accelerates the formation of bone trabeculae, and increases the bone tissue volume around the implant; in addition, MSCs significantly decrease the interference of a connective tissue component with osteogenesis and chondrogenesis.
